# Collagen and Discoidin Domain Receptor 1 Partnership: A Multifaceted Role in the Regulation of Breast Carcinoma Cell Phenotype

**DOI:** 10.3389/fcell.2021.808625

**Published:** 2021-12-22

**Authors:** Charles Saby, Erik Maquoi, Frédéric Saltel, Hamid Morjani

**Affiliations:** ^1^ Unité BioSpecT, EA7506, UFR de Pharmacie, Université de Reims Champagne-Ardenne, Reims, France; ^2^ Laboratory of Tumour and Developmental Biology, Groupe Interdisciplinaire de Génoprotéomique Appliqué (GIGA), Unit of Cancer, University of Liège, Liège, Belgium; ^3^ INSERM, UMR1053, BaRITOn Bordeaux Research in Translational Oncology, Bordeaux, France

**Keywords:** type I collagen, aging, DDRs, MT1-MMP, apoptosis, linear invadosomes, invasion, breast carcinoma

## Abstract

Type I collagen, the major components of breast interstitial stroma, is able to regulate breast carcinoma cell behavior. Discoidin domain receptor 1 (DDR1) is a type I collagen receptor playing a key role in this process. In fact, collagen/DDR1 axis is able to trigger the downregulation of cell proliferation and the activation of BIK-mediated apoptosis pathway. The aim of this review is to discuss the role of two important factors that regulate these processes. The first factor is the level of DDR1 expression. DDR1 is highly expressed in epithelial-like breast carcinoma cells, but poorly in basal-like ones. Moreover, DDR1 undergoes cleavage by MT1-MMP, which is highly expressed in basal-like breast carcinoma cells. The second factor is type I collagen remodeling since DDR1 activation depends on its fibrillar organization. Collagen remodeling is involved in the regulation of cell proliferation and apoptosis through age- and proteolysis-related modifications.

## Involvement of the Collagen/DDR1 Axis in Collagen-Induced Apoptosis

Many carcinomas and predominately breast cancers are characterized by a dense stroma associated with extensive type I collagen (COL1) deposition (Kalluri and Zeisberg, 2006). A hallmark of the malignant process is the acquisition of an invasive phenotype that allows cancer cells to breach their surrounding basement membrane, which is mainly composed of collagen IV and laminins (Liotta and Kohn, 2001). As a consequence, invading carcinoma cells become confronted with the 3D COL1-rich microenvironment of the reactive stroma. To develop metastatic capabilities, the invasive tumor cells must therefore adapt themselves to this structurally and biochemically distinct microenvironment (Hanahan and Weinberg, 2000; Hanahan and Weinberg, 2011).

To better understand the interplay between invading breast cancer cells and a COL1 dominated microenvironment, the phenotypes of different cell lines representing weakly invasive luminal-like (MCF-7 and ZR-75-1) and highly invasive basal-like (MDA-MB-231 and Hs578T) cells were investigated *in vitro* in three-dimensional collagen (3D COL1) gels (Maquoi et al., 2012). These 3D COL1 gels, which are characterized by the presence of self-assembled collagen fibers, represent a low rigidity and more physiologically relevant model than regular 2D plastic plates. Although the four cell lines proliferated well when seeded on tissue culture plates, they exhibited strongly divergent responses in 3D COL1. In line with their more mesenchymal phenotype, the COL1-embedded basal-like cell lines displayed a fibroblastic-like morphology characterized by the presence of elongated cytoplasmic protrusions. Dead or apoptotic cells were seldomly detected. In contrast, an extensive apoptosis was observed in the luminal-like cell lines, which exhibited plasma membrane blebbing as well as nuclear condensation and fragmentation. This apoptotic process was prevented upon treatment with Z-VAD-FMK, a pan caspase inhibitor. A transcriptomic analysis performed in luminal-like MCF-7 cells revealed that the exposure to 3D COL1 down-regulated the anti-apoptotic BCL-2 gene, while up-regulating different pro-apoptotic BCL-2 members, including BAD, BOK, and BIK (Assent et al., 2015). Among these, BIK was demonstrated to play a central role in the 3D COL1-induced apoptosis of the luminal-like cell lines (Maquoi et al., 2012; Saby et al., 2018). In contrast, 3D COL1 failed to induce BIK expression in the basal-like cell lines (Maquoi et al., 2012). It is worth noting that the COL1-dependent up-regulation of BIK expression in MCF-7 cells was only observed when the cells were fully embedded within a 3D COL1 gel and not when the cells were plated on top or covered by a 3D COL1 gel, suggesting that the sole presence of collagen fibers is not sufficient to induce this process. Conversely, embedding MCF-7 cells in 3D Matrigel [an extracellular matrix (ECM) mainly composed of basement membrane components] failed to promote apoptosis (Assent et al., 2015), demonstrating the requirement for COL1 in this process.

To decipher how 3D COL1 triggers apoptosis in the luminal-like cell lines, the expression of different cell-ECM adhesion molecules was compared in MCF-7 cells plated on 2D plastic plates or embedded within 3D COL1 gels. 3D COL1 down-regulated the mRNA level of different integrin family members (Assent et al., 2015), including ITGB1 and ITGA2, a major receptor for fibrillar collagens (Madamanchi et al., 2014; Bourgot et al., 2020). In contrast, the expression of discoidin domain receptor 1 (DDR1) was increased (Assent et al., 2015). A similar up-regulation of DDR1 by 3D COL1 was also reported in multipotent mesenchymal stem cells (Lund et al., 2009). At the protein level, the 3D COL1-dependent increase in DDR1 expression was associated with a down-regulation of the full-length receptor and a concomitant accumulation of a kinase-proficient membrane-anchored C-terminal fragment lacking the extracellular domains implicated in collagen interactions (Assent et al., 2015). DDR1 silencing or interfering with its tyrosine kinase activity by using pharmacological inhibitors prevented the induction the BIK-dependent apoptotic process in the luminal-like cell lines (Assent et al., 2015; Saby et al., 2018), supporting a critical role for DDR1 in 3D COL1-induced apoptosis. A similar pro-apoptotic function of DDR1 was demonstrated in HT-29 colon carcinoma cells encapsulated in 3D COL1 (Le et al., 2020). These observations contrast with the classical concept that DDR1 expression in cancer cells is associated with an increased cell survival and proliferation (for review, see Majo and Auguste, 2021). Interestingly, a reduction in DDR1 mRNA levels was shown in most middle- to high-grade human breast carcinomas compared with normal mammary tissues (Neuhaus et al., 2011). Low DDR1 expression tends to lead to a poor disease-free survival rate in triple-negative breast cancer patients (Koh et al., 2015) and is associated with poor prognosis in lung cancer patients (Ford et al., 2007). In contrast, high DDR1 expression is associated with a better relapse-free survival probability in breast cancer patients (Saby et al., 2019). DDR1 ablation in MMTV-PyMT mice, a transgenic model of luminal B breast cancer, accelerated tumor growth and lung metastasis (Takai et al., 2018). Collectively, these data support a potential cancer-protective function for DDR1 at least in breast and lung cancers. This protective role might stem, at least in part, from its capacity to promote the death of cancer cells confronted to COL1-rich microenvironments.

## Collagen Remodeling Regulates DDR1 Signaling and Collagen-Induced Apoptosis

In the case of breast carcinoma, COL1 density has been shown to be increased (Provenzano et al., 2008; Insua-Rodríguez and Oskarsson, 2016). Lysyl oxidase (LOX), which is known to play a role in enzymatic COL1 cross-linking and fibrils maturation, has been shown to be overexpressed in breast carcinoma and responsible for metastatic niche formation (Levental et al., 2009). Such alterations have been proposed to be used as a prognostic marker (Bredfeldt et al., 2014). At the topological level, COL1 fibrils alignment has been reported to limit protrusions and to enhance *breast* cancer cell *persistence* (Riching et al., 2014). Thus, such properties have been proposed to be used as prognostic signature for survival in human breast carcinoma (Conklin et al., 2011, 2018).

With a particularly long half-life of about 15 years in human soft tissues such as the skin (Verzijl et al., 2000), COL1 undergoes cumulative and irreversible non-enzymatic modifications which can lead to the generation of advanced glycation endproducts (AGE) (Simm et al., 2015). Among those AGE, pentosidine contributes to the formation of crosslinks between COL1 fibers that induces a disorganization of COL1 fibrils (Monnier et al., 2005; Van Gulick et al., 2019). Panwar and co-workers have reported that such alterations are able to induce changes in the mechanical properties of COL1 and its susceptibility to degradation by metalloproteinases (Panwar et al., 2015; 2018). The impact of age-related COL1 remodeling on cell behavior has been reported in several works. In lung carcinoma, aged COL1 has been shown to be responsible for a decrease of cell migration and invasion (Bartling et al., 2009). In prostate carcinoma cells, age-related changes of COL1 have been shown to increase cell proliferation (Damodarasamy et al., 2015). Interestingly, DDRs have been shown to be differentially activated in aged COL1. In fact, COL1 aging has been reported to impair discoidin domain receptor 2 (DDR2) activation and thus its cell growth suppressor effect in fibrosarcoma cells (Saby et al., 2016). In prostate carcinoma cells, DDR1 has been described as an anti-aging factor, which is able to regulate cell sensitivity to chemotherapies (Chappell et al., 2020).

**FIGURE 1:**
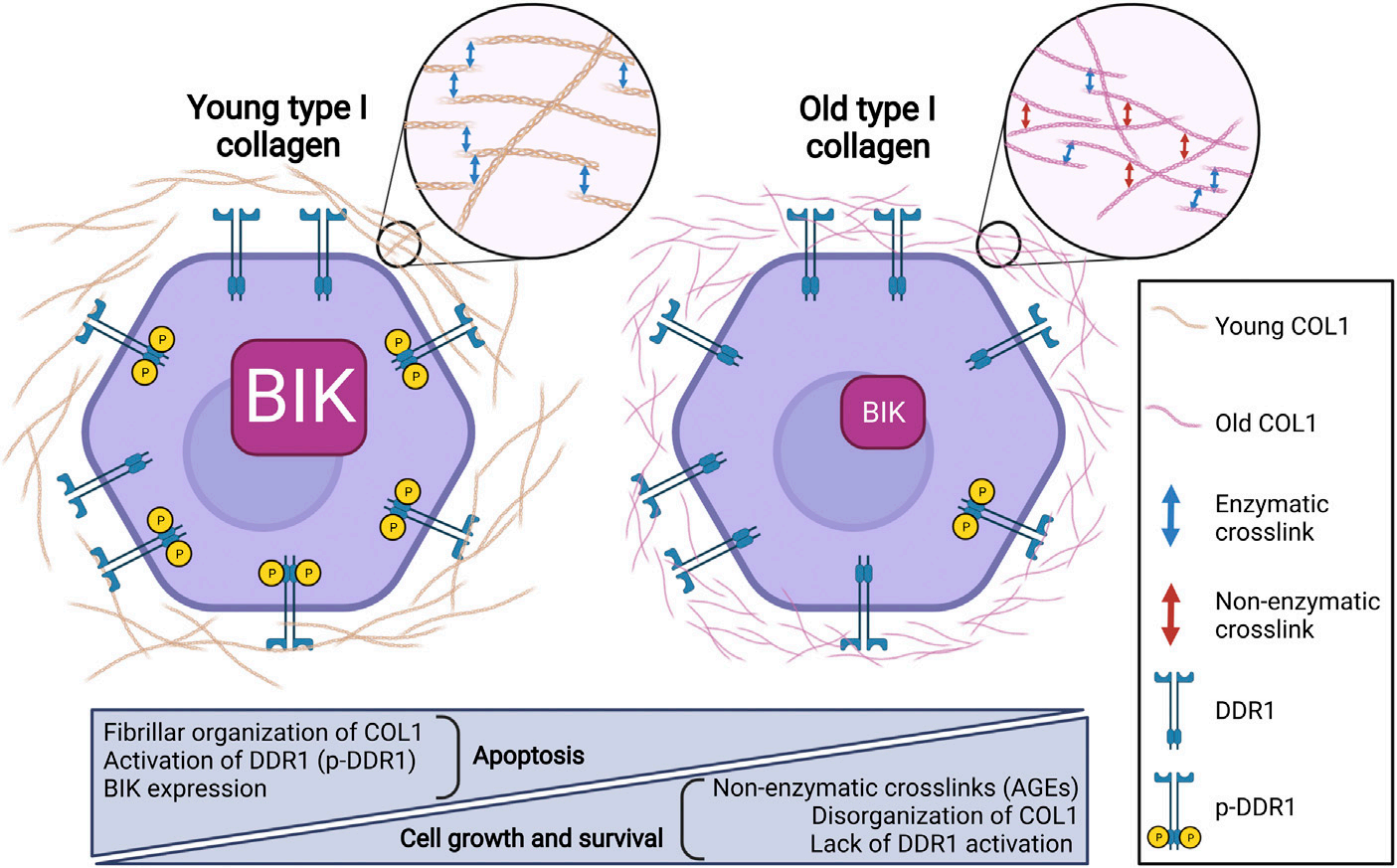
Collagen aging impairs DDR1-induced apoptosis by decreasing BIK expression in luminal breast carcinoma.

In order to better understand how the age-related modification of COL1 fibers could modulate the behavior of breast carcinoma cells in terms of proliferation and survival, we have analyzed such properties in two luminal breast carcinoma cell lines, MCF-7 and ZR-75-1 cells embedded in either young or old 3D COL1 matrices. Our results showed that both cell lines exhibited a lower cell growth in young COL1 when compared with the old one (Saby et al., 2018). Interestingly, DDR1 has been shown to be activated by 3D COL1 and to inhibit cell growth in these cells (Assent et al., 2015). We also showed that DDR1 depletion was able to promote cell growth in 3D young COL1, but not in the old one. To investigate whether a differential DDR1 activation by the two collagens was linked to cell growth regulation, DDR1 activation was analyzed. DDR1 was more phosphorylated in young 3D COL1 than in the old one, whereas DDR1 was expressed at the same level in these two 3D COL1 (Saby et al., 2018). These data were confirmed using nilotinib, a DDR1 tyrosine kinase function inhibitor. In fact, data showed that nilotinib was able to increase cell growth in 3D young COL1 to a level similar to that observed in the old one. Then, we decided to investigate whether the downregulation of cell growth in young 3D COL1 was associated to apoptosis, and thus to a downregulation of cell survival. Our results showed that COL1 aging promotes cell growth by impairing the DDR1/BIK signaling pathway that induced apoptosis in MCF-7 and ZR-75-1 cells. These observations were confirmed by knock-down and pharmacological inhibition of DDR1 in both luminal breast carcinoma cell lines. Both strategies led to a decrease in BIK expression and apoptosis in 3D young COL1 (Saby et al., 2018). Altogether, these results suggest that age-related COL1 alteration leads to a loss of the cell growth suppressor effect of 3D COL1, resulting from the impairment of the COL1-induced apoptosis which contributes to the growth and survival of luminal breast carcinoma cells (Figure 1). Thus, age-dependent structural modifications of COL1 may be considered when addressing the tumor behavior in elderly patients. This remodeling of the extracellular matrix of tumor stroma could therefore represent a potential therapeutic target.

Intra-tumoral heterogeneity is determined by the phenotypic and molecular diversity within the tumor and can be explained at least in part by cancer cell plasticity (Koren and Bentires-Alj, 2015). One of the key mechanisms that contributes to phenotypic plasticity and heterogeneity is the epithelial–mesenchymal transition (EMT). During EMT, cells can undergo dynamic transitions from an epithelial state, described by the presence of cell-to-cell junctions, interactions with basement membranes to a more mesenchymal phenotype, characterized by a fibroblast-like morphology linked with increased migratory and invasive properties (Nieto et al., 2016; Yang et al., 2020). Cells undergoing an EMT are known to be resistant to apoptosis. Consistent with this finding, BIK and DDR1 are both down-regulated during breast cancer-associated EMT (Taube et al., 2010). In breast cancer, this decrease of DDR1 expression has been linked to the increase of ZEB1 expression, which is involved in EMT, and which has been identified has a transcriptional repressor of DDR1 expression (Koh et al., 2015). Interestingly, DDR2 seems to also play a role in breast cancer EMT. Whereas DDR1 play a pro-apoptotic role, DDR2 has been shown to have a pro-metastatic role in breast carcinoma. In fact, studies from Longmore group has shown that DDR2 activation through COL1 interaction was able to stabilize SNAIL1, a transcription factor involved in the induction of EMT, thus leading to cancer cell migration and invasion (Zhang et al., 2013). The same group has later shown that in an *in-vivo* mouse model of breast carcinoma, inhibition of DDR2 by the small-molecule allosteric inhibitor of DDR2 extracellular domain (WRG-28), was able to disrupt DDR2/COL1 interaction, thus leading to a decrease in SNAIL1 activity and an inhibition of DDR2 pro-metastatic effect (Grither and Longmore, 2018). Interestingly, in those papers, DDR2 signaling was induced during EMT, but was not necessary for the induction of EMT (Zhang et al., 2013; Majo and Auguste, 2021). Together, these data suggest a switch of DDRs expression during EMT.

Membrane-type 1 matrix metalloproteinase (MT1-MMP, MMP-14), a membrane-anchored MMP is typically expressed by stromal cells as well as cancer epithelial cells undergoing EMT (Pulyaeva et al., 1997). This proteinase is a potent matrix-degrading enzyme able to cleave COL1 (Ohuchi et al., 1997), as well as a number of cell surface-associated proteins including DDR1 (Fu et al., 2013). This capacity to cleave two major actors of the 3D COL1-induced apoptosis, prompted us to investigate the potential roles of MT1-MMP during this process.

**FIGURE 2:**
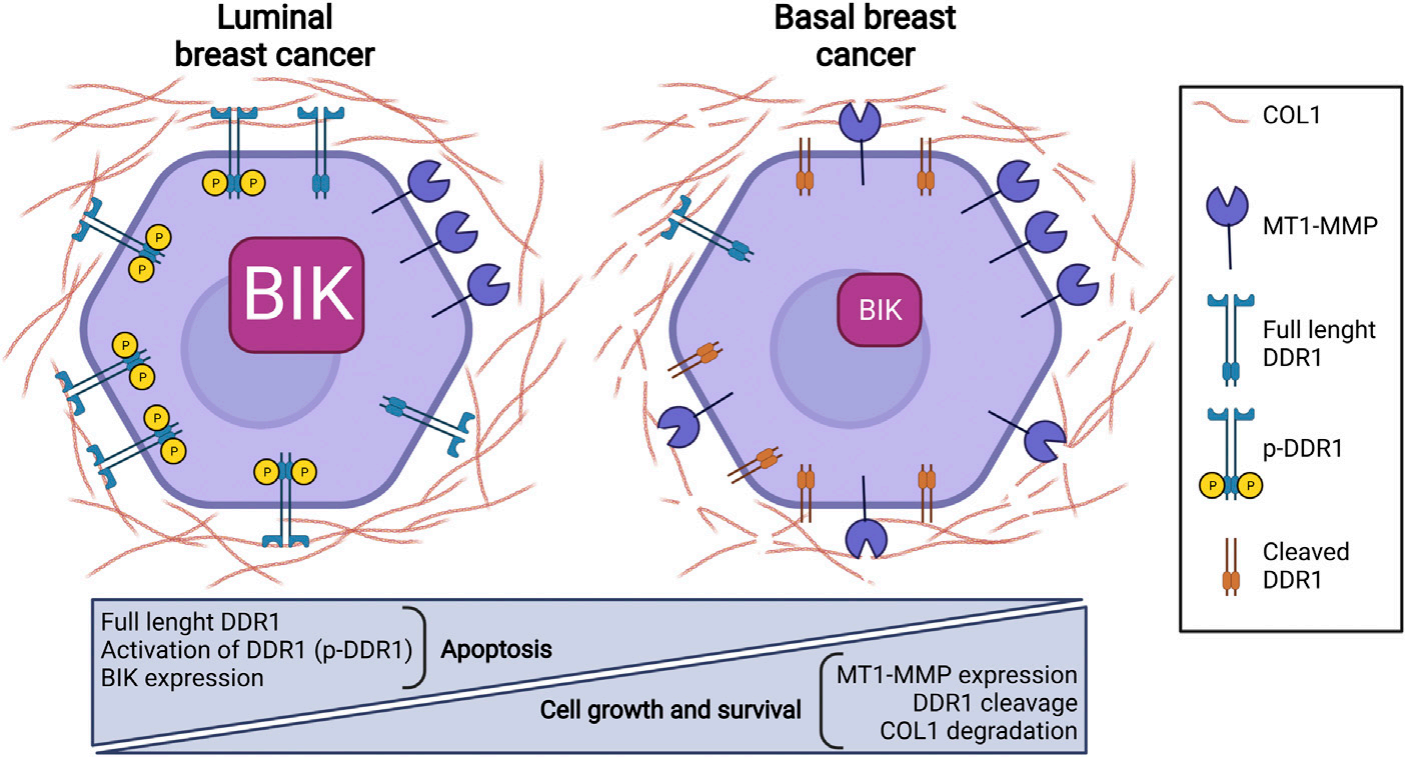
MT1-MMP expression impairs DDR1-induced apoptosis in basal breast cancer cells.

To decipher how MT1-MMP could modulate 3D COL1-induced apoptosis, shRNA strategy against MT1-MMP mRNA was used in the MDA-MB-231 basal-like breast carcinoma cell line, which displays a mesenchymal phenotype characterized by high expression of MT1-MMP and low level of DDR1 (Saby et al., 2019). MT1-MMP silencing partially restored full length DDR1 expression and phosphorylation, leading to a decreased cell growth associated with a restored 3D COL1/DDR1/BIK axis-mediated apoptosis (Saby et al., 2019). Similarly, the overexpression of DDR1 in MDA-MB-231 cells suppressed cell growth by restoring COL1/DDR1/BIK1-induced apoptosis, suggesting that the low expression level of full length DDR1 observed in basal-like breast carcinoma confers them with a capacity to resist collagen-induced cell growth suppression and apoptosis. Interestingly, the combination of MT1-MMP depletion and DDR1 overexpression synergistically increased 3D COL1-induced apoptosis to a level similar to that observed in luminal breast carcinoma MCF-7 cells (Assent et al., 2015; Saby et al., 2018). Collectively, these results suggest that the induction of MT1-MMP expression combined with DDR1 downregulation/degradation during the acquisition of mesenchymal features play an important role in the aggressiveness of basal breast cancer cells and could accordingly be considered as a potential prognosis biomarker (Figure 2) (Saby et al., 2019).

## Implication of Collagen-DDR1 Interaction in Linear Invadosomes Formation and Tumor Invasion

More and more articles report the involvement of DDR1 and/or DDR2 in cancer and tumor invasion (Leitinger, 2014; Henriet et al., 2018; Majo and Auguste, 2021). This trend has been confirmed for several years and reinforces the idea that these receptors are interesting as molecular targets to limit tumor progression. These receptors have been first described as collagen receptors, including COL1 as an important component of ECM involved in tumor progression.

In this context, invadosomes are actin-based structures present in invasive tumor cells and associated with ECM degradation and thus with cancer cell invasion and metastasis (Cambi and Chavrier, 2021; Lin et al., 2021). These invadosomes can be organized in different forms, in a rosette or in isolated points. It is known that in breast cancer COL1 deposition increases, and it has been demonstrated that only fibrillar organization of COL1 can organize and induce invadosomes formation. Indeed, COL1 promote formation of linear invadosomes along fibrils, and such organization is associated with an important increase of cell capacity to degrade the surrounding ECM including COL1 itself (Juin et al., 2012). In breast cancer, linear invadosomes are dependent on the DDR1 receptor, particularly in breast cancer cells harboring mesenchymal or basal-like phenotype such us MDA-MD-231 cells. DDR1 activation through collagen stimulation allows the recruitment of the actin polymerization machinery and in particular the Rho GTPase Cdc42 and its GEF Tuba (Juin et al., 2014). In this study, authors demonstrated that linear invadosomes can be formed in 3D COL1 matrix and that DDR1 depletion using DDR1 RNAi decrease breast cancer cell ability to invade 3D COL1 gel.

DDR2 is also involved in breast tumor invasion by inducing mesenchymal phenotype, invasion, and metastasis (Ren et al., 2014; Barcus et al., 2021). Thus, it should be interesting to investigate the implication of this receptor in the formation of linear invadosomes. This is even more important since some breast tumors express DDR2 or both receptors. Currently, the only study reported on DDR2 and linear invadosome concern endothelial cells. It is known that DDR2 can be involved in tumor progression through the tumor neo-angiogenesis process (Abou Hammoud et al., 2020). In fact, the ECM degradation is necessary for the formation of new vessels in this process. Thus, endothelial cells are also able to form linear invadosomes and to contribute to the formation of new vessels, and consequently to tumor progression by a process which is dependent on DDR2.

From the mechanistic point of view, it will also be important to better define the role of DDR1 and DDR2 in the formation of invadosomes and invasion, in order to deeply understand their involvement in cell adhesion and ECM degradation. It will also be important to investigate more precisely the molecular mechanisms associated to these processes. In addition, it will be necessary to analyze the formation of linear invadosome in a more complex model in which the other components of the microenvironment could be present, such as cytokines (TGF-beta), metalloproteinase (MMP14) or COL1 crosslinking agent lysyl oxidase (LOX) (Ezzoukhry et al., 2016). All this information may provide therapeutic strategies to inhibit tumor cell dissemination and tumor growth in breast carcinoma.
